# RNA Editing-Dependent and -Independent Roles of Adenosine Deaminases Acting on RNA Proteins in Herpesvirus Infection—Hints on Another Layer of Complexity

**DOI:** 10.3390/v15102007

**Published:** 2023-09-27

**Authors:** Vlatka Ivanišević, Lidia Žilić, Marina Čunko, Hana Fadiga, Ivana Munitić, Igor Jurak

**Affiliations:** Department of Biotechnology, University of Rijeka, 51000 Rijeka, Croatialidia.zilic@student.uniri.hr (L.Ž.);

**Keywords:** herpesvirus, ADAR, dsRNA, RNA editing, miRNA, latency, innate immunity

## Abstract

The Adenosine Deaminases Acting on RNA (ADAR) catalyze the posttranscriptional deamination of adenosine residues to inosine in double-stranded RNAs (dsRNAs, A-to-I editing), preventing the overactivation of dsRNA sensor molecules and interferons. RNA editing is the cornerstone of innate immunity that distinguishes between self and non-self (virus), and it is essential for normal regulation of cellular homeostasis. Although much is already known about the role of ADAR proteins in RNA virus infection, the role of ADAR proteins in herpesvirus infection remains largely unexplored. In this review, we provide several lines of evidence from studies of different herpesviruses for another level of complexity in regulating the already intricate biphasic life cycle of herpesviruses.

## 1. Introduction

Viruses depend on the host cell for replication and constantly evolve in response to host defenses. Various host pattern recognition receptors (PRRs, e.g., Toll-like receptors (TLRs), retinoic acid-inducible gene 1 (RIG-I)–like receptors, protein kinase R (PKR), interferon-gamma-inducible protein 16 (IFI16), etc.) recognize the so-called pathogen-associated molecular patterns (PAMPs), such as viral capsids, surface glycoproteins, and viral genomes and transcripts, and trigger signaling cascades that lead to the establishment of the replication nonpermissive state or initiate cell death (reviewed in [1,2,3]). Although viruses respond to host defenses with their countermeasures (i.e., inhibitors of these signaling pathways), the defense mechanisms and activation of downstream effectors must be precisely balanced to prevent overactivation and damage to the host.

Members of the Adenosine Deaminase Acting on RNA (ADAR) protein family catalyze the conversion of adenosines to inosines (A-to-I editing), one of the most common forms of posttranscriptional RNA modification, and provide the molecular mark that distinguishes host RNA from foreign RNA (e.g., viral). They suppress the hyperactivation of dsRNA sensors (such as PKR, RIG-I/MDA5 (melanoma differentiation-associated protein 5), OAS (2′-5′-oligoadenylate synthetase)/RNase L, and ZBP1 (Z-DNA-binding protein 1, also known as DNA-dependent activator of IFN-regulatory factors (DAI)) that could lead to autoimmunity (reviewed in [4,5,6]). On the one hand, A-to-I editing of RNA transcripts can affect multiple cellular processes through various mechanisms, including mRNA translation, splicing, RNA structure, and RNA silencing, and on the other hand, it attenuates dsRNA sensing in an editing-dependent and/or -independent manner (reviewed in [7]). Viruses may benefit from all of these mechanisms, and there is ample evidence to support this assumption. This review presents all currently published studies on ADAR-mediated posttranscriptional modifications and the potential role of ADARs in herpesvirus infection, major dsDNA viruses that have been largely neglected in A-to-I studies. The activity of ADAR affects several aspects of gene expression and regulation of replication in various herpesviruses, from modulation of miRNA biogenesis and miRNA targeting to complete alteration of the biological properties of edited transcripts. In addition, there are several lines of evidence that ADAR proteins are required for efficient reactivation and replication of herpesviruses. Studies of diverse and evolutionarily distant herpesviruses, e.g., from human viruses to viruses of mollusks, suggest the fundamental importance of ADAR for replication of these viruses. The role of the ADAR proteins remains largely unexplored, but it is clear that A-to-I editing adds another level of complexity to the biology of herpesviruses.

## 2. The ADAR Protein Family in Brief

**Figure 1:**
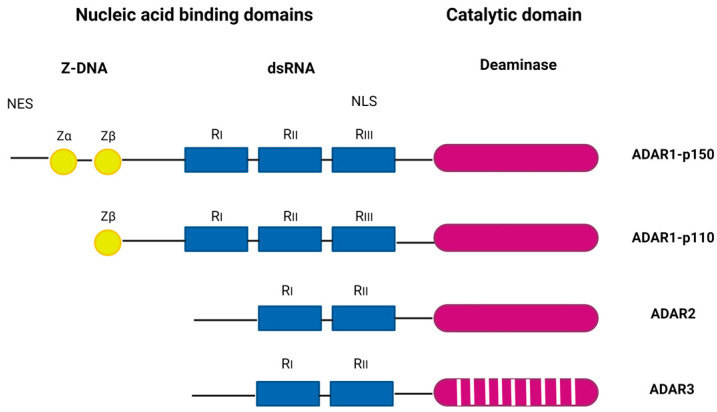
The ADAR protein family. ADAR1, interferon-inducible p150 and constitutively expressed p110 form, ADAR2, and ADAR3. ADAR1 and ADAR2 have deaminase activity. ADAR3 lacks deaminase activity and has a role in regulation of editing. Z-DNA binding domains (Zα and Zβ) are shown as yellow circles, the dsRNA binding domains (R_I_, R_II_, and R_III_) are shown as blue rectangles, and deaminase catalytic domains are shown are purple ovals. A stripped purple oval indicated a lack of deaminase activity. Figure adapted from [11] and generated in BioRender.com (Available online: https://www.biorender.com/, accessed on 10 September 2023).

ADAR proteins catalyze the C6 deamination of adenosine (A) to inosine (I) in double-stranded RNA (dsRNA), one of the most common posttranscriptional modifications of RNAs, known as A-to-I editing (reviewed in [8]). Inosine forms base pairs with cytosine (C) instead of uracil (U), which alters the structure, stability, biogenesis, and coding of transcripts. The ADAR family includes three genes: ADAR1 (ADAR), ADAR2 (ADARB1), and ADAR3 (ADARB2) (Figure 1). ADAR1 encodes the constitutively expressed ADAR1 p110, which is localized in the nucleus, and the interferon-inducible ADAR1 p150, which is localized in the cytoplasm and nucleus. ADAR1 is the major RNA editor in humans, and most of its hundreds of millions of editing sites have been found in Alu elements, many of which are in introns and 3′-untranslated regions [9,10]. 

ADAR1 p150 has two Z-DNA binding domains, in contrast to p110 which has only one, and shows a broader range of targets compared to p110 [12]. ADAR1 is ubiquitously expressed and is inducible by many viruses, including human cytomegalovirus (HCMV) [13], Kaposi’s sarcoma-associated virus (KSHV) [14], reoviruses [15], etc. In mice, ADAR1 deficiency is embryonically lethal [16], and in humans, dysregulations of ADAR1 activity are associated with a number of diseases, including Aicardi–Goutières syndrome, an autoimmune disorder and interferonopathy [17]; bilateral striatal necrosis dystonia [18]; dyschromatosis symmetrica hereditaria, a skin pigmentation disorder [19]; and various cancers [20]. The main role of ADAR1 editing is to prevent sensors (PKR, RIG-I/MDA5, LGP2, ZBP1, and OAS/RNase L) from recognizing endogenous dsRNA as non-self and to prevent hyperactivation of downstream signaling [21,22,23,24,25]. In addition, ADAR1 forms a complex with a number of different proteins, including Dicer, to promote miRNA processing and RNA-induced gene silencing [26].

In humans, ADAR2 is highly expressed in the brain, arteries, lungs, and bladder and is responsible for site-specific editing (reviewed in [27]). The major physiological function of ADAR2 is attributed to exonic RNA editing of the neuronal glutamate receptor (GluR), a process essential for neuronal homeostasis [28]. In contrast to ADAR1 and ADAR2, the expression of ADAR3 is mainly restricted to the nervous system, particularly the hippocampus and amygdala [29]. Although ADAR3 has two copies of dsRNA-binding domains it does not exhibit catalytic deaminase activity and has an editing regulatory function [30]. Recently, ADAR3 was also shown to regulate MAVS (mitochondrial antiviral signaling; the common downstream adaptor for a number of dsRNA sensors) expression, suggesting that its role extends beyond regulation of editing [31]. In mice, genetic ablation of ADAR3 does not lead to embryonic lethality, but mice lacking ADAR3 show impaired short- and long-term memory [32].

The role of ADAR proteins in viral infections has been studied for a great number of RNA and a few DNA viruses, and proviral and antiviral roles have been demonstrated. These studies are discussed in detail elsewhere (reviewed in [11,33]), and we will focus only on recent discoveries on herpesviruses below.

## 3. Herpesviruses in Brief

Members of the order *Herpsesvirales* (herpesviruses) are widespread in nature and have been discovered in a variety of animals. The order includes three distinct virus families: (a) the *Orthoherpesviridae* (formerly the *Herpesviridae*; i.e., the herpesviruses of mammals, birds, and reptiles), (b) the *Alloherpesviridae* (the herpesviruses of fish and amphibians), and (c) the *Malacoherpesviridae* (the herpesviruses of mollusks). Herpesviruses are large dsDNA viruses that share several biological properties, including the establishment of latent infection as a mechanism for lifelong persistent infection and transmission [34,35]. The replication cycle of herpesviruses entails two phases, the lytic (i.e., productive) phase and the latent phase. During the lytic phase, viral genes are abundantly expressed in a tightly regulated temporal cascade of gene expression. In the second phase of infection, herpesviruses reach target cells in which they initiate a latent infection program. In general, viral genomes take the form of closed circular and chromatinized molecules, from which only a small subset of specific genes is expressed, and no viral progeny are produced. Each herpesvirus establishes latency in a specific group of cells, which may be distinct from the cells in which the virus replicates productively. Importantly, latent genomes retain the ability to reactivate and generate new infectious virions during the lytic phase. The molecular mechanisms of establishment, maintenance, and reactivation from the latency phase vary widely among different herpesviruses but clearly represent an exceptionally successful mechanism for dissemination and persistence in nature [35]. Herpesviruses have co-evolved with their hosts and developed numerous specific adaptations to host defense mechanisms, such that cross-species infections are rare in nature [35,36]. Malacoherpesviruses are a notable exception, as both OsHV-1 and HaHV-1 can infect multiple species, including distantly related species such as oysters and clams [37].

### The Orthoherpesviridae

The family Ortoherpesviridae encompasses viruses that infect mammals, birds and reptiles and includes nine clinically important human herpesviruses (human herpesvirus 1–5, HHV-6A and -6B, and HHV-7 and HHV-8). Based on different biological properties the family is divided into three subfamilies: Alphaherpesvirinae, Betaherpesviriane, and Gammaherpesvirinae. Alphaherpesviruses have relatively broad host range, rapid productive phase, and establish latent infection in neurons, primarily in sensory ganglia. This subfamily contains viruses isolated from numerus different animals including important human pathogens herpes simplex virus 1 (HSV-1, also HHV-1) and HSV-2 (HHV-2) and varicella zoster virus (VZV, HHV-3). Betaherpesviruses have slow productive cycle and have very restricted host range. Usually, betaherpesviruses infect various cell types and tissues and establish latency in CD34+ hematopoietic progenitor cells and CD14+ monocytes or T lymphocytes. This subfamily contains numerous viruses including HCMV (HHV-5), human herpesvirus 6A and 6B (HHV-6A and HHV-6B), human herpesvirus 7 (HHV-7), murine cytomegalovirus (MCMV), rat cytomegalovirus (RCMV), etc., grouped in several genera. Gammaherpesviruses include human onocoviruses, Epstein–Barr virus (EBV, or HHV-4), and KSHV (HHV-8). Latency is usually established in lymphoid tissues in either B or T lymphocytes. Interestingly, while alpha- and betaherpesviruses primarily enter productive phase in infected cells in culture, gammaherpesviruses predominate in latent phase [34,35].

## 4. ADAR and Herpesviruses

### 4.1. Evidence for ADAR-Mediated RNA Editing in Alphaherpesviruses

#### 4.1.1. Herpes Simplex Virus 1 (HSV-1)—Latent miRNA Editing

HSV-1 is an important human pathogen, a widely distributed and extensively studied virus known to cause cold sores. After productive infection in the mucosal epithelium in the oro-labial or genital area, HSV-1 reaches nearby neurons and migrates via axons to cell bodies in sensory ganglia, where it initiates latent infection. During latency, no viral progeny are produced, and only transcripts derived from the latency-associated transcript (LAT) locus are abundantly expressed [38]. LATs are multiple non-coding RNAs and precursors of miRNAs and stable introns of 2 kb and 1.5 kb. The exact function of LATs remains unclear, but their functions in suppression of gene expression in the productive phase, inhibition of apoptosis, and chromatin remodeling have been demonstrated [39,40,41,42,43]. HSV-1 expresses miRNAs from more than 20 loci (miR-H1–miR-H18 and miR-H26-H29), and often both strands of the miRNA duplex can be detected, indicating efficient utilization of the coding potential [44,45,46,47,48]. Most HSV-1 miRNAs were detected in productive infections, whereas only a small subset of miRNAs (miR-H2-H8) was detected in latently infected human ganglia [46,49]. Many HSV-1 miRNAs are conserved in HSV-2 and are in antisense to transcripts encoding important viral proteins. For example, miR-H2, -H7, and -H2 are encoded opposite ICP0, an important viral transcriptional coactivator and regulator of innate immunity, suggesting their function in repressing these genes [44,50,51,52,53]. Apart from this, the functions of HSV-1 miRNAs are still poorly defined.

The complexity of miRNA regulation of HSV-1 infection was recently further increased by the discovery of posttranscriptional editing of HSV-1 miRNAs in the latency phase. Zubković et al. found that miR-H2-3p, but not other miRNAs encoding HSV-1, is hyperedited in latently infected human and mouse ganglia, suggesting functional significance at this stage of infection [49,54]. miR-H2-3p is edited within the seed sequence that determines binding specificity for transcripts targeted for regulation, and thus editing expands its targeting potential. In an overexpression system, the edited miR-H2-3p was found to have the potential to regulate ICP4 (an essential viral protein) in addition to ICP0, which is encoded in the opposite direction to this miRNA [54]. At present, it is not clear which ADAR protein is responsible for the editing phenomenon or what biological significance the editing has for HSV-1 replication. Nevertheless, these results suggest that other non-coding RNAs abundant in latently infected neurons, such as the LAT intron, may also be edited and that editing may play an important role in the biology of these RNAs.

#### 4.1.2. Varicella Zoster Virus (VZV)—Edited Novel Viral Transcripts

VZV is known to cause chickenpox (varicella) and shingles (zoster). VZV is a highly infectious virus transmitted by inhalation of aerosolized virions released from skin lesions. VZV initially infects mucosal cells in the upper respiratory tract, where it is recognized by dendritic cells and transported to lymph nodes, where infection spreads to T lymphocytes, which shed the virus into the skin. Latency is established in ganglia throughout the neuroaxis. The reactivated virus migrates anterograde to the dermatome where it can cause herpes zoster [55]. Latent VZV, similar to HSV-1, expresses only a few restricted transcripts, including ORF63 and the spliced latency-associated VZV transcript (VLT), which is in antisense position to the gene ORF61, an important viral transactivator similar to HSV-1 ICP0 [56]. Despite the obvious similarity in control of latency between HSV-1 and VZV, VZV is the only human herpesvirus for which no encoded miRNAs have been identified.

In contrast to HSV-1, the phenomenon of RNA editing of VZV transcripts was studied in its lytic phase. Using long-read nanopore sequencing technology of RNA extracted from lytic VZV infections, Prazsak et al. identified a number of novel VZV transcripts, including several transcripts derived from loci near the origin of replication (oriS), termed nroRNA (near-replication-origin) transcripts [57]. One of these transcripts, NTO3, expressed antisense to ORF62 and showed a very high frequency of A-to-G substitutions that were not present in the overlap of other transcripts, suggesting a hyperediting event of this transcript. The authors suggest that editing of NTO3 ensures the thermodynamic stability of the RNA secondary structure and that editing may have an impact on the potential regulation of the antisense transcript ORF62; however, this remains to be investigated.

#### 4.1.3. Gallid Herpesvirus 2 (GaHV-2, or Marek’s Disease Virus 1 (MDV-1)

GaHV-2 is an oncogenic virus that infects chickens causing major losses in poultry production. GaHV-2 induces aggressive T-cell lymphomas in only a few weeks leading to the death of the chicken. The genome of the virus is colinear with HSV-1, including internal repeats that contain a number of important genes (e.g., viral telomerase subunit vTR and meq, the major oncogene), latency-associated transcript long non-coding RNA (LAT lncRNA), and three clusters of miRNAs [58,59,60].

Interestingly, Figueroa et al. recently identified another 5′-caped polyadenylated, alternatively spliced lncRNA, named ERL lncRNA [61]. The transcript is encoded within the repeat flanking the unique long region of the genome overlapping with the transcripts of R-LORF5a, meq, and 14D genes and two clusters of miRNAs, mdv1-miR-M9-M4 and -M11-M1. In contrast to LAT lncRNA, which is a hallmark of latency, the ERL lncRNA displayed similar levels during primary productive infection, lytic reactivation, and latency. Moreover, ERL lncRNA displayed hyperediting (i.e., more than 25% of its transcripts) during primary productive infection, but not during reactivation from latency or in latency [61]. Although the function of the ERL lncRNA and the biological significance of its editing are unknown, the authors have suggested that this transcript may play a role as a natural antisense transcript regulating the two miRNA clusters.

Taken together, current information on roles of ADAR in alphaherpesviruses is very limited. However, the above-mentioned studies clearly show that editing affects transcripts of these viruses, and functional studies are yet to be conducted.

### 4.2. Evidence of ADAR-Mediated RNA Editing in Betaherpesviruses—Edited Host miRNA

HCMV is a ubiquitously distributed virus that infects up to 60–100% of adults. HCMV is the leading cause of congenital disease and an important and life-threatening opportunistic pathogen in immunocompromised individuals, especially transplant recipients [62]. HCMV establishes latency primarily in CD34+ hematopoietic progenitor cells (HPCs), and differentiation of these cells into dendritic cells (DC) or macrophages in response to cytokine and growth factor signaling leads to virus reactivation [63]. The hallmark of HCMV biology is modulation of the host immune response, which includes a large number of viral proteins and gene products that interfere with antigen presentation (e.g., viral miR-UL112 downregulates the activating NK ligand MICB and reduces killing of infected cells) [64,65].

To date, the role of ADAR in HCMV infection has been investigated in only one case, yet the study provided several important findings. Nachmani et al. found that ADAR1 p110, but not p150, is significantly upregulated in cells productively infected with HCMV in culture [13]. Interestingly, they did not observe the same upregulation in other dsDNA (HSV-1, HSV-2, and adenovirus) or RNA viruses (influenza virus A and human metapneumovirus). It appears that HCMV increases the expression of the ADAR1-p110 isoform by activating one of the four promoters (1B) that regulate ADAR1 gene expression. The increased level of ADAR1 protein was accompanied by an increased rate of the edited form of host miR-376a (miR-376a(e)) compared with mock-infected cells, i.e., from 50% in mock-infected cells to approximately 80% in HCMV-infected cells. Editing of miR-376a occurs in the seed region of this miRNA, resulting in a drastic change in target selection, including loss of regulation of a key NK cell-activating receptor MICB. On the other hand, the edited miRNA gained the ability to downregulate HLA-E, the ligand of two immune receptors, the inhibitory heterodimer CD94/NKG2A, and the activating CD94/NKG2C, facilitating NK cell cytotoxicity and immunelimination of HCMV-infected cells. Taken together, on the one hand, the virus uses host and viral miRNAs (miR-UL112 and a number of host miRNAs, including miR-376a) to reduce the elimination of infected cells, and on the other hand, the induced editing miR-376a renders cells susceptible to NK cell toxicity. Although seemingly contradictory, it is quite puzzling which of these activities is dominant and relevant to a particular phase of dynamic viral replication, i.e., from maintenance of latency to reactivation and dissemination [13].

This study did not address the possible editing of viral transcripts or viral miRNAs. However, in a study with a similar virus, murine CMV (MCMV), no evidence of edited MCMV miRNAs was found [66].

### 4.3. Gammaherpesviruses: Editing-Dependent and Editing-Independent Roles of ADAR Proteins

#### 4.3.1. Epstein–Barr Virus (EBV, HHV-4)—Editing Affects miRNA Biogenesis

EBV is a ubiquitous human virus that infects more than 90% of the adult population and causes no disease in the vast majority of healthy carriers [67]. However, EBV can induce B lymphocyte proliferation in vivo and has been linked to a number of cancers, including Burkitt’s lymphoma, Hodgkin’s disease, nasopharyngeal carcinoma, and gastric cancer (reviewed in [68]). Recently, a large longitudinal study of a cohort of more than 10 million young adults found that the risk of developing multiple sclerosis increases 32-fold after infection with EBV [69]. EBV establishes its latency predominantly in B cells, which is divided into five patterns depending on the expression of the viral latency gene expression (latency 0, I, IIa, IIb, and III). In addition, EBV encodes more than 20 miRNAs located in two clusters, the BART and BHRF1 regions of the genome, which are involved in regulating the transition from the lytic to the latent phase and in attenuating the antiviral response [70].

The precursors of several of these miRNAs, including pri-miR-BHRF1-1, pri-miR-BART3, pri-miR-BART6, pri-miR-BART8, pri-miR-BART11, and pri-miR-BART16, have bene found posttranscriptionally edited at specific sites in cells latently infected with EBV [71,72]. The editing frequency of one of these pri-miRNAs, pri-miR-BART6, reached as high as 50–70% of all transcripts. Importantly, editing pri-miR-BART6 severely impairs the processivity of Drosha (the type III RNase that binds the dsRNA hairpin region of pri-miRNA and initiates the first step of miRNA biogenesis that generates pre-miRNAs [73]) and greatly reduces the amount of mature miR-BART6. Moreover, miR-BART6 targets Dicer (also RNase III, which cleaves pre-miRNAs into short double-stranded miRNAs that produce mature miRNAs), and consequently, processing of its pri-miRNA modulates not only Dicer levels but also global repression by miRNAs [71]. Modulation of Dicer levels may play a role in regulating latency and balancing the on–off switch for reactivation [71]. In a similar study, pri-miR-BART3 was also found to be hyperedited and to affect Drosha processing and miRNA targeting of Dicer by editing the seed region of miRNA [72]. However, several studies did not find edited mature forms of EBV miRNAs, suggesting that the levels of edited miRNAs are low or exhibit rapid turnover [74]. To date, it remains puzzling which member of the ADAR protein family, ADAR1 or ADAR2, edits EBV pri-miRNAs. However, based on the low expression of ADAR2 in cells harboring latent EBV, it is suggested that ADAR1 p110 may play a predominant role [71].

In addition to miRNA studies, massive RNA editing was observed in lncRNA transcripts derived from the EBV origin of replication, oriPtL and oriPtR, which have a role in promoting productive gene expression and DNA replication [75]. These newly identified late viral transcripts maintained their localization within the nucleus and were predicted to form long hairpin structures that provide a configuration for recognition by RNA editing enzymes. Indeed, using immunoprecipitation assays, the authors demonstrated the direct interaction of ADAR1 and oriP transcripts during induced reactivation. Interestingly, oriP transcripts were found in association with the paraspeckle complex assembly factor NONO, suggesting a role of these transcripts in modulating the antiviral stress response [75]. However, it is not known whether RNA editing is required for this interaction.

#### 4.3.2. Kaposi’s Sarcoma-Associated Herpesvirus (KSHV, HHV-8)

KSHV is associated with at least three human malignancies: Kaposi’s sarcoma (KS), primary effusion lymphoma (PEL), and multicentric Castleman’s disease (MCD) [76]. KSHV infection in tumors and established PEL cell lines is predominantly latent, and only a limited number of genes are expressed including latency-associated nuclear antigen 1 and 2 (LANA), v-FLIP, K15, K12, and miRNAs [77]. K12 RNA is the most common transcript present during latent phase of viral replication of KSHV and encodes three Kaposin proteins (Kaposin A, B, and C) and miR-K10 that retains tumorigenic activity [78,79,80].

- KSHV—RNA Editing Phenomena

Sequence heterogeneity and potential editing phenomena in herpesviruses were first noted for KSHV [14,81,82], and only recently several studies on KSHV have shed light on the importance and diverse functions of ADAR proteins in herpesvirus infection. In their remarkable study providing the first evidence for herpesvirus-encoded miRNAs, Pfeffer at al. found A-to-G sequence variation within KSHV-encoded miR-K12-10, and suggested posttranscriptional editing [81]. Such editing would also affect the Kaposin A protein, which is encoded from the same locus with the amino acid glycine replaced by a serine. Indeed, the editing of Kaposin mRNA was later confirmed in two PEL cell lines using a sensitive PCR and Northern blot assays [83]. Remarkably, editing of K12 transcript, which includes changes within both Kaposin A and miR-K10, eliminates entirely its transforming activity, i.e., ability to induce focus formation in transfected cells or produce tumors in nude mice [83]. Moreover, levels of K12 editing increases dramatically with reactivation and lytic progression [14,83], suggesting that its tumorigenic activity is required for maintenance of latency. In addition, authors demonstrated that recombinant ADAR1 made highly specific edits to K12 transcript in vitro, suggesting that ADAR1 was responsible for editing of this transcript in PEL cell lines [83]. Nonetheless, the surge in the levels of Kaposin transcript editing is not concomitant with the increase in ADAR1 [84], but it correlates with increased RNA binding during reactivation [84]. Recently Rajendran et al. performed comprehensive analysis of the KSHV editome landscape in different cells and identified numerous additional editing spots in viral transcriptome, some of which are conserved in all tested cells, including editing within Kaposin, LANA and RTA transcript (amino acid change E378G), and pri-miR-K12-4 (two sites within the loop and one within the seed sequence) [84]. Similar to Kaposin transcript, editing of pri-miR-K12-4 was abolished in cell devoid of ADAR1 expression. Interestingly, editing of pri-miR-K12-4 had a strong impact on Drosha processivity resulting in reduced levels of mature miRNAs [84]. This result was consistent with the finding that depletion of ADAR1 resulted in increased levels of mature miR-K12-4. Furthermore, authors demonstrated that editing within the seed region not only expands the repertoire of potential RNA that can be targeted but also switches ontological association of potential targets. For example, targets of unedited miR-K12-4-3p are ontologically enriched for processes involved in transcription, metabolic processes, and macromolecule biosynthesis, while edited miR-K12-4-3p targets are enriched for cell growth and development [84]. Although biological relevance of miR-K12-4-3p editing remains elusive, it is intriguing that it is required for infectious virion production [84], and that A-to-I editing may contribute to efficient KSHV infection.

- KSHV—Editing-Independent Roles of ADAR

Herpesvirus infections, including KSHV, are known to trigger innate immune responses by activating host DNA and RNA sensors (e.g., TLRs, RIG -I, MDA5, and cGAS, IFI16), leading to the induction of type I interferons and proinflammatory cytokines [85,86]. On the other hand, the main role of ADAR1 is to mark RNAs as self and prevent overactivation of these signaling pathways. Therefore, it can be predicted that ADAR1 may play a pro-herpesvirus role and that its deficiency may limit efficient herpesvirus infection. Indeed, in their recent work, Zhang et al. have shown that ablation of ADAR1 (both forms p150 and p110) in cells latently infected with KSHV strongly inhibits induced reactivation [87]. Moreover, they show that knockdown of ADAR1 increases IFN production and induces a variety of antiviral genes during KSHV reactivation, suggesting that ADAR1 is required for optimal reactivation of KSHV. Nevertheless, many ADAR1-activated pathways could be responsible for the observed induction of IFN. To address this question, Zhang et al. carefully examined potential molecular pathways in cells lacking ADAR1 and showed that depletion of RIG -I and, to a lesser extent, MDA5 rescued KSHV reactivation and abrogated RLR singling and activation of TBK1 and IRF3, ultimately leading to reduced induction of IFN. Depletion of MAVS in ADAR1-deficient cells rescued KSHV reactivation and additionally confirmed their conclusions [87]. The exact trigger for activation of RIG -I in reactivating cells remains unknown, but Zhang et al. show evidence of enhanced binding of RIG -I to dsRNA in cells lacking ADAR1. To our knowledge, this is the first study to show an editing-independent role for ADAR1 in herpesvirus infection, underscoring the immense importance of this protein. It is reasonable to assume that other herpesviruses also rely on the presence of ADAR proteins in cells and that similar paradigms apply within the same host species. However, different herpesviruses have evolved different adaptations to host defenses, so the need for a specific ADAR-mediated pathway may differ.

### 4.4. The Malacoherpesviridae–Herpesviruses of Mollusks

Herpesviruses of invertebrates are largely unknown, limiting our understanding of the diversity and evolution of nonmammalian, as well as mammalian, herpesviruses. Osterid herpesvirus-1 (OsHV-1) and Haliotid herpesvirus-1 (HaHV-1), which infect marine bivalves and abalones (sea snails), respectively, are the only two invertebrate herpesviruses isolated to date [88]. However, there are sequence evidence for several new viruses in the family, suggesting a wide diversity of these viruses [37]. They can cause detrimental infections in aquaculture species and significant economic losses in oyster and abalone farming. Unfortunately, studies on malacoherpesviruses are largely limited to infections in vivo and are therefore very challenging.

Nonetheless, using RNA analysis, Rosani et al. demonstrated the upregulation of ADAR1 homolog in the bivalve Crossostrea gigas and in the gastropod Haliotis diversicolor supertexta durign OsHV-1 or HaHV-1 infection, respectively, which correlated with the extensive hyperediting of viral and host RNAs [89,90]. The ADAR hyperediting on the OsHV-1 transcripts occurred in genomic hot spots characterized by the presence of overlapping genes on the opposite strand. Similarly, Bai at al. showed upregulation of ADAR1 homolog in the bivalve Scapharca broughtonii infected with OsHV-1 and in Haliotis diversicolor supertexta infected with HaHV-1 [91]. Although the kinetics of ADAR1 upregulation were somewhat different between these two species, hyperediting increased with time in both infections, reaching maximal levels late in infection. Important to note, in C. gigas, ADAR1 transcript was also upregulated after stimulation with poly(I:C), which correlated with hyperediting of a number of genes involved in antiviral response, miRNA maturation, and epigenetic regulation [90]. In addition, several lines of evidence led authors to speculate that levels of editing might directly differentiate between oysters resistant and susceptible to virus infection.

Table 1 lists all currently available studies on editing phenomenon and roles of ADAR proteins in herpesvirus infection.

## 5. Discussion

Over the years and through intensive studies, much has been learned about the biology and pathogenesis of herpesviruses, which has not only improved understanding but also enabled treatment of disease and improvement of health. The role of ADAR proteins in dsDNA viruses, including herpesviruses, is a rather neglected area of research. This is somewhat understandable considering that ADARs are dsRNA-binding proteins and that most studies have focused on RNA viruses. The goal of this literature review is to provide clues to another level of herpesvirus complexity that has not been adequately explored. We focus exclusively on the potential role of the ADAR proteins, although other RNA editing proteins, such as the activation-induced cytidine deaminase/apolipoprotein B mRNA-editing enzyme catalytic polypeptide-like (AID/APOBEC) protein family also play important roles (reviewed in [92]).

A-to-I editing (editome) in herpesvirus infections has been studied in detail in only a few cases. However, current evidence suggests that a substantial amount of editing of viral transcripts occurs during productive and latent infection in all viruses studied. While in RNA viruses editing could affect the genome, contribute to genome diversity, and impact fitness (hypermutations) and pathogenesis of the virus (reviewed in [7,33]), such scenarios are unlikely in dsDNA viruses, and the focus is on editing of coding and non-coding transcripts. It is important to note that increased editing rate usually correlates with increased expression of ADAR proteins, but differential expression of ADAR proteins has only been studied for some herpesviruses. Nonetheless, some important conclusions can be drawn. First, productive infection (HCMV, OsHV-1, and HaHV-1) [13,89,90,91], including the reactivation process (KSHV) [87], triggers the expression of ADAR proteins, but not for all herpesviruses (HSV-1 and HSV-2) [13]. Second, HCVM selectively activates only the p110 promoter but not the interferon-inducible p150 [13], whereas KSHV induces both forms [14]. These results strongly suggest that different viruses use different mechanisms and may have different requirements for A-to-I editing at different phases of infection. For example, hyperediting of one of HSV-1-encoded miRNAs (miR-H2-3p) has been found in latently infected human ganglia, and to much lower extent in productively infected cells in culture, clearly indicating a potential importance of this process for latent infection [54]. One may hypothesize that the lower extent of editing in productive infection compared to latent infection is simply a matter of accessibility. On the other hand, KSHV transcripts and miRNAs are edited to a lesser extent during latency and increase during reactivation. Current evidence indicates that ADAR does not randomly edit viral transcripts, but specifically edits multiple transcripts (e.g., Kaposin) and a selection of pri-miRNAs to alter the properties of these gene products and generate different cellular environments required by the virus [83]. This is probably only one of the mechanisms, but the dramatic loss of Kaposin transformation activity strongly suggests its importance.

In herpesviruses, the functional consequences of editing are best seen in miRNAs. Editing of both KSHV and EBV miRNA precursor transcripts (pri-miRNAs) negatively affects Drosha processivity, limiting the amounts of these miRNAs and deregulating their direct targets [71,72,84]. Moreover, EBV miR-BART3 and –BART6 directly regulate Dicer, so editing of these miRNAs has a global effect on the RNA-interreference (RNAi) system [71,72]. Whether the precursor of hyperedited HSV-1 miR-H2 inherits impaired processivity from Drosha is not known, but 50% rate of miR-H2 editing suggests that it does not [49,54]. Furthermore, there is evidence that edited miR-H2 is loaded onto Ago as efficiently as non-edited miRNA, in contrast to edited miR-BART6 where editing also suppresses RISC loading [54]. It is important to mention that editing-mediated alterations of miRNA biology are not unique to viruses, rather well noted for cellular miRNAs (i.e., editing effects biogenesis, loading, re-targeting, and stability of miRNAs).

In addition to transcripts encoding miRNAs, there are other transcripts with dsRNA structure that have been found edited in VZV, Ga- HV -2, KSHV, OsHV-1, and HaHV-1 infections, but clear evidence for a relevant biological role is much less obvious [57,75,84,90]. It is important to note that ADAR1 has recently been shown to be a potent regulator of circular RNAs (circRNAs) [93,94], a class of covalently closed-circular single-stranded RNAs implicated in several human diseases (reviewed in [95,96,97,98]). circRNAs are also encoded in several herpesviruses and differentially expressed during infections and in different tissues [99]. Although their roles in infection remain to be elucidated, their remarkable stability suggests that ADARs and RNA editing may also affect the biogenesis and function of viral circRNAs.

Editing-(in)dependent functions of ADAR. The role of ADAR1, primarily ADAR p150, in suppressing antiviral signaling has been demonstrated for several RNA viruses (MeV, VSV, IV, HCV, CoV, etc.) [7]. However, it is not clear whether A-to-I editing is always required for suppressive function or whether it depends only on dsRNA binding and interaction with RNA sensors. There is ample evidence that the absence of ADAR1 p150 leads to overactivation of PKR (e.g., MeV, VSV, EMCV, and HIV), but in some viruses other sensing pathways take over (e.g., the OAS pathway in CoV and RIG -I in IV), and sometimes activation of PKR can even be beneficial to the virus (HCV) [7,100]. Therefore, it is very interesting that ADAR1 mediates the immunosuppression required for efficient reactivation via the MDA5 pathway during KSHV reactivation [87]. It is now known whether other sensing pathways are involved in this process. It will be very exciting to learn whether the ADAR1/MDA5 axis is also required for other herpesviruses or whether other pathways are more important.

Some open questions still need to be discussed. The studies described have provided us with only a small glimpse of the possible role of ADAR proteins and the complexity they bring to herpesvirus infection. There is still a large gap in basic knowledge about the editome in infected cells. For example, editing of many viruses, including HSV1 and HSV-2, is unknown, and the dynamics of editing during reactivation are unexplored for most viruses. Editing can affect the processing (biogenesis), stability, interactome, and function of RNAs, thereby uncovering editing will contribute to a better understanding of the basic biological properties of transcripts and their functions. The contribution of the two ADAR proteins, ADAR1 and ADAR2, to a variety of replication strategies, latency niches, and hosts remains to be better defined. In addition, how expression of the ADAR proteins is regulated during infection remains to be better understood (e.g., KSHV and HCMV induce ADAR1 expression during productive infection but with different protein forms, whereas HSV-1 does not affect ADAR1 levels).

On the other hand, a body of evidence suggests that ADAR p150, the interferon-stimulated form of ADAR, is solely responsible for suppressing cytoplasmic sensors (PKR, RIG -I/MDA5, ZBP1, and OAS) and attenuating their antiviral response. However, which signaling pathways are involved (different in different herpesviruses? do all signaling pathways contribute?)? what are the exact triggers of the signaling pathways? how these triggers link ADAR1 and dsRNA sensors? what is a role of various posttranscriptional and posttranslational modifications? and many other questions remain to be addressed.

Finally, no herpesvirus gene product has yet been identified that mimics or directly affects the functions of ADAR, such as the VAI transcript of AdV [101], the NS1 protein of influenza virus [102], and the E3L protein of vaccinia virus [103].

Overall, the biology of herpes viruses has been studied in great detail, but there is still much to learn, and the complexity of these viruses always offers new surprises.

## Figures and Tables

**Table 1:** Known functions of ADAR proteins in herpesvirus infection.

Virus Taxonomy			Virus	ADAR Activity	Ref.
***Herpesvirales***	*Ortoherpesviridae*	*Alphaherpesvirinae*	HSV-1 (HHV-1)	ADAR1 expression levels maintained during productive infection. Editing of HSV-1 miR-H2-3p in latency and to lesser extent in productive infection. Function: increased targeting repertoire of miR-H2-3p.	[13,49,54]
			VZV (HHV-3)	Dynamics of ADAR expression levels: unknown. Editing of lncRNA NTO3 (antisense to *ORF63*). Function: unknown.	[57]
			GaHV-2	Dynamics of ADAR expression levels: unknown. Editing of ERL lncRNA. Function: unknown.	[61]
		*Betaherpesvirinae*	HCMV (HHV-5)	ADAR1 p110 is upregulated in productive infection. Editing of host miR-376a. Function: edited miRNA gains specificity to downregulates HLA-E and abolishes targeting of MICB (ligand of activating NKG2D receptor), facilitating elimination of HCMV infected cells.	[13]
		*Gammaherpesvirinae*	EBV (HHV-4)	Dynamics of ADAR expression levels: unknown. Editing of pri-BHRF1-1, pri-miR-BART3,-BART6, -BART8, -BART11, and -BART16. Editing of vlncRNA oriPtL and oriPtR. Functions: affected Drosha processing of pri-miR-BART6 and -BART3 resulting in lower levels of miRNAs, and loss of posttranscriptional regulation of their targets (Dicer). miR-BART3 seed sequence editing abolished Dicer targeting. Functions of edited oriPtL and oriPtR: unknown.	[71,72,75]
			KSHV (HHV-8)	ADAR1 expression levels maintained from latent to lytic infection. * ADAR1 (all forms) increased during reactivation. * Editing of K12 transcript, LANA, RTA, etc., and pri-miR-K12-10, pri-miR-K12-4 Functions: Editing eliminates K12 transforming activity and reduces pri-miR-K12-4 processing by Drosha. Increased repertoire of miR-K12-4 targets. ADAR1 prevents activation of RIG-I signaling and enables efficient virus reactivation.	[14,81,83,84,87]
	*Malacoherpesviridae*		OsHV-1 HaHV-1	ADAR1 upregulated in productively infected host. Editing: increased global editing of viral and host transcripts during infection. Function: unknown.	[89,90,91]

* Studies show conflicting results.
